# Seed/catalyst-free growth of zinc oxide nanostructures on multilayer graphene by thermal evaporation

**DOI:** 10.1186/1556-276X-9-83

**Published:** 2014-02-18

**Authors:** Nurul Fariha Ahmad, Nurul Izni Rusli, Mohamad Rusop Mahmood, Kanji Yasui, Abdul Manaf Hashim

**Affiliations:** 1 Malaysia-Japan International Institute of Technology, Universiti Teknologi Malaysia, Jalan Semarak, Kuala Lumpur 54100, Malaysia; 2 School of Electrical System Engineering, Universiti Malaysia Perlis, Kuala Perlis, Perlis 02000, Malaysia; 3 Faculty of Electrical Engineering, Universiti Teknologi MARA, Shah Alam, Selangor 40450, Malaysia; 4 Department of Electrical Engineering, Nagaoka University of Technology, Kamitomioka-machi, Nagaoka, Niigata 940-2137, Japan; 5 MIMOS Berhad, Technology Park Malaysia, Kuala Lumpur 57000, Malaysia

**Keywords:** Graphene, Thermal evaporation, Zinc oxide, Nanostructure, Hybrid integration

## Abstract

We report the seed/catalyst-free growth of ZnO on multilayer graphene by thermal evaporation of Zn in the presence of O_2_ gas. The effects of substrate temperatures were studied. The changes of morphologies were very significant where the grown ZnO structures show three different structures, i.e., nanoclusters, nanorods, and thin films at 600°C, 800°C, and 1,000°C, respectively. High-density vertically aligned ZnO nanorods comparable to other methods were obtained. A growth mechanism was proposed based on the obtained results. The ZnO/graphene hybrid structure provides several potential applications in electronics and optoelectronics.

## Background

In recent years, strong attentions have been paid in the growth of semiconductor nanostructures on graphene [1-5] for electronic and optoelectronic applications. Nanostructures such as nanowires, nanorods, nanoneedles, nanosheets, and nanowalls can offer additional functionality to graphene for realizing advanced nanoscale applications in photovoltaics, nanogenerators, field emission devices, sensitive biological and chemical sensors, and efficient energy conversion and storage devices [6-8]. This is due to the superb properties of nanostructures such as high aspect ratio, extremely large surface-to-volume ratio, and high porosity [6-10]. Graphene has a great potential for novel electronic devices because of its extraordinary electrical, thermal, and mechanical properties, including carrier mobility exceeding 10^4^ cm^2^/Vs and a thermal conductivity of 10^3^ W/mK [11-14]. Therefore, with the excellent electrical and thermal characteristics of graphene layers, growing semiconductor nanostructures on graphene layers would enable their novel physical properties to be exploited in diverse sophisticated device applications. Graphene is a 2D hexagonal network of carbon atoms which is formed by making strong triangular σ-bonds of the *sp*^2^ hybridized orbitals. This bonding structure is similar to the (111) plane of zinc-blende structure and C plane of a hexagonal crystalline structure. With this regard, the growth of semiconductor nanostructures and thin films on graphene is feasible. Recently, there are several works on the growth and application of graphene/semiconductor nanocrystals that show desirable combinations of these properties not found in the individual components [15-20].

The 1D zinc oxide (ZnO) semiconducting nanostructures are considered to be important multifunctional building blocks for fabricating various nanodevices [21,22]. Since graphene is an excellent conductor and transparent material, the hybrid structure of ZnO/graphene shall lead to several device applications not only on Si substrate but also on other insulating substrates such as transparent glass and transparent flexible plastic. Owing to the unique electronic and optical properties of ZnO nanostructures, such hybrid structure can be used for sensing devices [23-25], UV photodetector [26], solar cells [27], and light-emitting diodes [28]. ZnO nanostructures have been synthesized by various physical and chemical growth techniques [23]. These techniques include thermal evaporation [5,29], hydrothermal [2,3] and electrochemical deposition [4], and metal-organic vapor-phase epitaxy (MOVPE) [1]. In this paper, we report the seed/catalyst-free growth of ZnO structures on multilayer (ML) graphene by thermal evaporation. The dependence of substrate temperatures on the properties of grown structures was studied. Based on the obtained results, a growth mechanism was proposed.

## Methods

**Figure 1:**
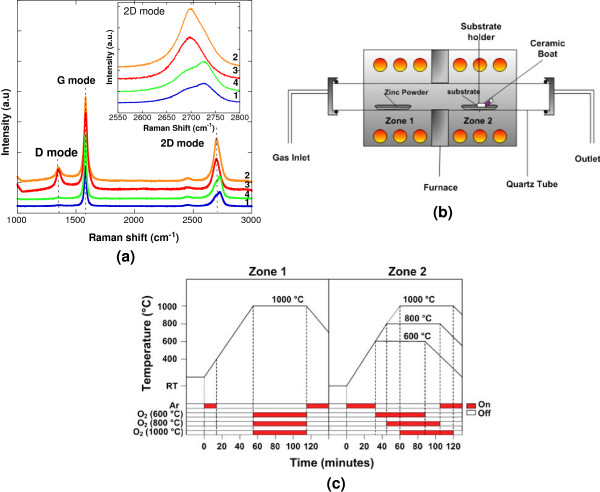
Raman spectra of ML graphene (a), schematic of growth setup (b), and growth time chart (c).

A ML graphene on SiO_2_/Si (Graphene Laboratories Inc, Calverton, NY, USA) was used as a substrate. Figure 1a shows the measured Raman spectra of the ML graphene. The 2D peaks at approximately 2,700 cm^-1^ of the Raman spectra for graphite as shown by locations 1 and 4 have broader and up-shifted 2D band indicating few layer graphene [30]. Figure 1b shows the schematic of the experimental setup. The growth was carried out by thermal evaporation technique in dual zone furnace. High-purity metallic Zn powder (99.85%) and oxygen (O_2_) gas (99.80%) were used as the sources. Prior to the growth process, the substrate was treated with organic cleaning of ethanol, acetone, and deionized (DI) water to remove any unwanted impurities on the substrate. Zn powder of approximately 0.6 g was spread evenly into a ceramic boat. The ceramic boat was placed in the zone 1 of the furnace, while the substrate was placed inclined at 45° in the zone 2 of the furnace. The distance between source and substrate was fixed at 23 cm. Two independent temperatures were applied to the furnace system. Here, T1 denotes to the set temperature (ST) of the source while T2 denotes to the ST of the substrate. Firstly, the temperature of zone 2 was raised to T2 (i.e., 600°C, 800°C, or 1,000°C) in argon (Ar) environment (Ar flow rate of 200 sccm). Then, the temperature of zone 1 was raised to T1 (1,000°C). The flow of Ar was stopped when the temperature of zone 1 reached 400°C (Zn melting point, 419°C). This was done in order to avoid the transfer of Zn particles to substrate prior to actual growth. The heating of Zn powder was continued until it reached 1,000°C. It was confirmed from several attempts that such high temperature was needed for continuous and constant evaporation of Zn. After reaching 1,000°C, O_2_ (400 sccm) was introduced for 1 h of growth time. Finally, the furnace was turned off and the samples were cooled down to room temperature. Figure 1c summarizes the growth procedures. The as-grown ZnO was examined using field-emission scanning electron (FESEM) microscopy (SU8030, Hitachi, Chiyoda, Tokyo, Japan), dispersive X-ray (EDX) spectroscopy, X-ray diffraction (XRD) (Bruker, AXES, D8 Advance, Bruker Corporation, Billerica, MA, USA) and photoluminescence (PL) spectroscopy (Horiba JobinYvon, Tokyo, Japan).

## Results and discussion

**Figure 2:**
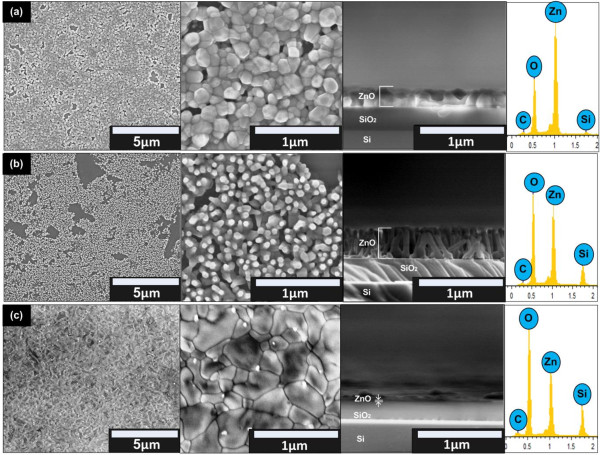
**FESEM images and EDX spectra of grown ZnO. (a)** 600°C. **(b)** 800°C. **(c)** 1,000°C.

Figure 2a,b,c shows the FESEM images and EDX spectra of the grown ZnO structures on graphene at temperatures of 600°C, 800°C, and 1,000°C, respectively. The surface morphology of the grown ZnO strongly depends on the substrate temperature. From the surface and cross-sectional images, it can be seen that the grown ZnO structures show three different morphologies, i.e., nanocluster, nanorod, and thin film structures at 600°C, 800°C, and 1,000°C, respectively. As shown by the EDX spectra, only Zn, O, Si, and carbon (C) elements were detected in all samples. The total compositional atomic percentages of Zn and O for the as-grown structures were found to be 87% for 600°C and 80% for both 800°C and 1,000°C. However, the composition ratio of Zn atoms to O atoms in samples decreases with the increase of temperature where the ratio is found to be 0.55, 0.33, and 0.23 for temperatures of 600°C, 800°C, and 1,000°C, respectively. This result shows that the nucleation of Zn particles is less promoted at high temperature. It is speculated that such tendency may be due to the formation of large etch pit and less horizontal nucleation which is explained in the growth mechanism. Detection of C element confirmed the presence of graphene on SiO_2_/Si substrate and was not etched away during the growth process. The calculated density of nanorods for samples grown at 800°C was estimated to be around 6.86 × 10^9^ cm^-2^ which is relatively high and comparable to other synthesis techniques either on graphene [1,2] or Si substrate [29]. Table 1 summarizes the density, diameter, length, and average aspect ratio of the grown ZnO including comparison with other works.

**Table 1:** Density, diameter, length, thickness, and average aspect ratio of the grown ZnO structures

	**Temperature (°C)**	**Density (cm**^**-2**^**)**	**Diameter of nanorods/nanoneedles (nm)**	**Length of nanorods (nm)**	**Thickness (nmn**	**Average aspect ratio**
This work	600	-	-	-	~200	-
	800	6.86 × 10^9^	50-150	200-380	-	2.85
	1,000	-	-	-	~60	-
[1]	400	4 × 10^9^	100 ± 10	1,000 ± 100	-	10.0
	600	8 × 10^7^	90 ± 20	4,000 ± 600	-	44.4
	750	5 × 10^7^	-	3,500 ± 500	-	-
[29]	800	1.2 × 10^8^	200-500	-	-	-

**Figure 3:**
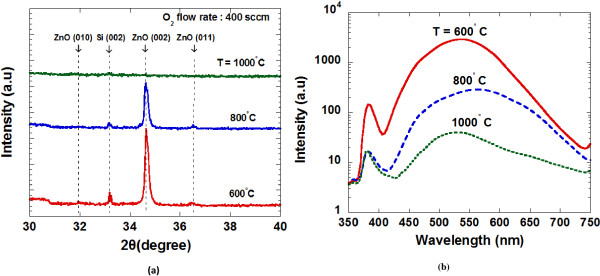
XRD (a) and PL spectra (b) of grown ZnO structures.

Figure 3a shows the measured XRD spectra for the sample grown at different substrate temperatures. The as-grown ZnO at 600°C and 800°C exhibit hexagonal wurtzite structure indicated by the presence of prominent peak at approximately 34.46° corresponding to ZnO (002) diffraction peak. A relatively high intensity of this peak indicates that the preferred growth orientation of the as-grown ZnO is towards the *c*-axis and it is consistent with the FESEM image shown in Figure 2. A very weak peak, approximately at 36.4° corresponding to ZnO (011) diffraction peak, was also observed in samples grown at 600°C and 800°C. However, no prominent peak of ZnO was observed for the sample grown at 1,000°C due to the very thin thickness of the grown layer.

The optical characteristics of ZnO were investigated using room temperature (RT) PL spectroscopy. Figure 3b shows the measured PL spectra for the samples grown at substrate temperatures of 600°C, 800°C, and 1,000°C. Here, two distinct peaks were observed. The first peak approximately at 383 nm for sample grown at 600°C and 382 nm for the samples grown at 800°C and 1,000°C were observed in the UV region. As reported, the dominant peaks at the UV region are attributed to the near-band edge emission (NBE) or recombination of free exciton [29,31]. The peaks in the visible region appear approximately at 534, 561, and 525 nm for the samples grown at 600°C, 800°C, and 1,000°C, respectively. The strong peak in the visible region, i.e., green emission is associated with specific defects such as O vacancies and Zn interstitials and these defects are responsible for the recombination of the green luminescence [31,32]. The highest peak intensity in UV emission and green emission was observed for the sample grown at 600°C. A small PL blueshift by 1 nm in the UV emission has been observed in the sample at 800°C. This may be due to the shape transitions to the well-faceted hexagonal structure [29]. The intensity of green emission peak seems to decrease with the increase of temperature. It is well reported that the crystallinity of the grown structure by vapor-phase method improves with the increase of temperature [32]. Low structural defects such as O vacancies and Zn interstitials may give sharper and stronger UV emission and weaker green emission [33]. However, measurement of low-temperature PL is required to obtain more accurate and precise information about the crystallinity of the grown ZnO structures.

**Figure 4:**
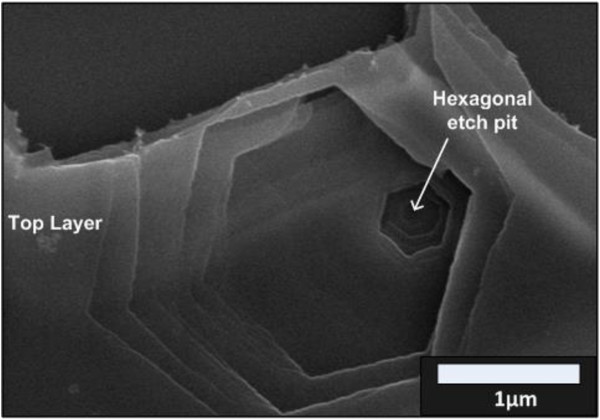
FESEM image of hexagonal etch pit of ML graphene.

**Figure 5:**
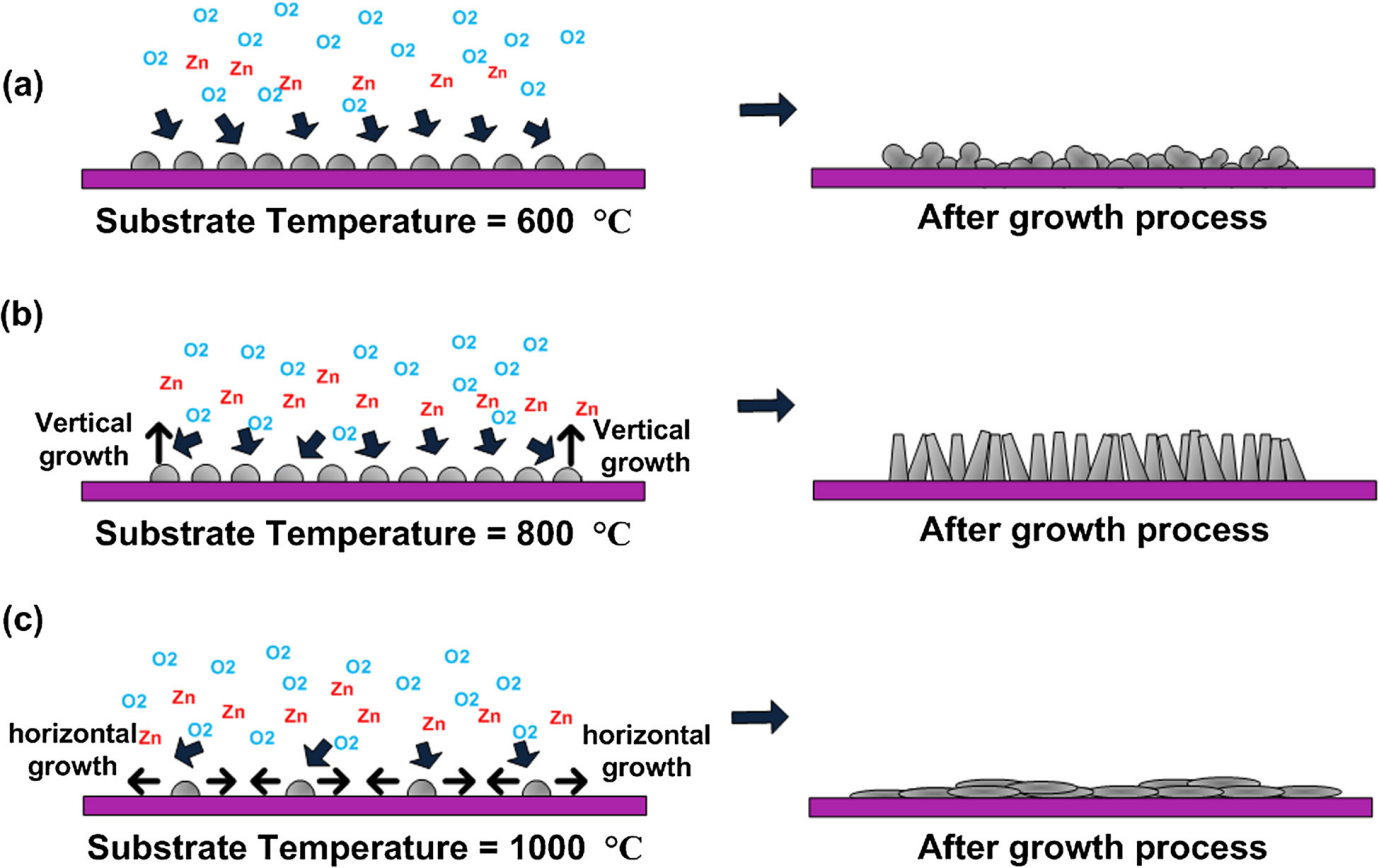
**Growth mechanism of ZnO structures on graphene at substrate temperatures. (a)** 600°C. **(b)** 800°C. **(c)** 1,000°C.

It was reported that C-C bonding of graphene can be broken by heating at high temperature of 600°C in O_2_ ambient, leading to the formation of etch pit [34]. Figure 4 shows the SEM image of hexagonal etch pit of multilayer graphene at 800°C for 10 min in O_2_ environment. It is speculated that the nucleation rates of Zn on the graphene strongly depend on the breaking rates of C-C bonds of graphene. Figure 5a,b,c illustrates the growth mechanism of ZnO structures on graphene at substrate temperatures of 600°C, 800°C, and 1,000°C, respectively. As shown in Figure 5a, the breaking of C-C starts to take place once O_2_ gas is introduced. Since the substrate temperature is low (600°C), the breaking rates can be considered to be low, resulting to less nucleation of Zn particles on graphene or in other words, less formation of Zn-C bonds. This results to the formation of ZnO nanoclusters or nanodots. However, the breaking of C-C bonds increases with the growth time and thus resulting to the increase in nucleation of Zn particles, thus promoting the formation of ZnO nanoclusters. Since the substrate temperature of 600°C is considerably low, the vertical growth of ZnO on ZnO nanoclusters seems to be low. As shown in Figure 5b, when the substrate temperature is increased to 800°C, the breaking rates of C-C bonds increase, resulting to the increase in the nucleation of Zn particles. As the growth time increases, the nucleation of Zn particles increases and thus, resulting to the increase in formation of ZnO nanoclusters. At the same time, since the substrate temperature is high, the vertical growth of ZnO on the ZnO nanoclusters seems to be well promoted. This can be seen in Figure 2b where there is a high density of nanorods grown at temperature of 800°C. As shown in Figure 5c, when the temperature is further increased to 1,000°C, the breaking rates of C-C bonds seem to be extremely high, resulting to highly dense larger etch pits. After the bonding of Zn and C at the surrounding of etch pit has been completed, the subsequent bonding of Zn and O tends to take place in horizontal direction rather than vertical direction. It is speculated that the direct bonding of Zn and O on SiO_2_ seems to be difficult to happen. Therefore, the bonding has to be induced laterally from the edge of etch pit in order to fully cover the etched area. As a result, such behavior of ZnO nucleation in the horizontal direction leads to the formation of ZnO thin film. This can be seen in Figure 2c where continuous thin film was formed.

## Conclusions

The effects of substrate temperatures on the morphological and optical properties of the grown ZnO on ML graphene were studied. Substrate temperatures seem to be a dominant parameter in determining the morphologies of ZnO structures since it is able to promote the breaking rates of C-C bonds of graphene. Based on the obtained results, the growth mechanism was proposed and discussed.

## Competing interest

The authors declare that they do not have any competing interests.

## Authors’ contributions

NFA designed and performed the experiments, participated in the characterization and data analysis of FESEM, EDX, XRD, and PL, and prepared the manuscript. NIR participated in the data analysis and preparation of manuscript. MRM participated in the PL characterization. KY participated in the revision of the manuscript. AMH participated in the monitoring the experimental work, data analysis, discussion, and revision of the manuscript. All authors read and approved the final manuscript.
